# The complete mitochondrial genome of *Palomena viridissima* (Hemiptera: Pentatomidae) and phylogenetic analysis

**DOI:** 10.1080/23802359.2021.1909442

**Published:** 2021-04-08

**Authors:** Wen-Ting Chen, Li-Jun Zhang, Ya Cao, Ming-Long Yuan

**Affiliations:** a State Key Laboratory of Grassland Agro-Ecosystems, Lanzhou University, Lanzhou, China; b National Demonstration Center for Experimental Grassland Science Education, Lanzhou University, Lanzhou, China; c College of Pastoral Agricultural Science and Technology, Lanzhou University, Lanzhou, China; d Ministry of Agriculture and Rural Affairs, Key Laboratory of Grassland Livestock Industry Innovation, Lanzhou, China

**Keywords:** Insects, mitochondrial DNA, Pentatominae, phylogenetic analysis

## Abstract

Here, we sequenced and annotated the complete mitochondrial genome (mitogenome) of *Palomena viridissima* (Hemiptera: Pentatomidae). This mitogenome was 15,118 bp long, comprising of 13 protein-coding genes (PCGs), 22 transfer RNA genes (tRNAs), 2 ribosomal RNA genes (*rrnL* and *rrnS*) and a large non-coding control region. The *P. viridissima* mitogenome with an A + T content of 76.0%, presented a positive AT-skew (0.11) and a negative GC-skew (-0.13). Ten PCGs started with a typical ATN codon, two PCGs started with TTG (*atp8*, *nad1*), whereas the remaining one used AAC (*cox1*). All tRNAs had a typical secondary cloverleaf structure, except for *trnS1* which lacked the dihydrouridine arm. The Bayesian phylogenetic analysis based on mitogenomic data supported a sister relationship of *P. viridissima* and *Nezara viridula* from the same tribe Nezarini and recovered a phylogeny of Pentatominae: (Menidini + (Strachiini + (Pentatomini + ((Cappaeini + Halyini) + (Eysarcorini + (Nezarini + Carpocori)))))).

Pentatominae (Insecta: Heteroptera: Pentatomidae), consisting of approximate 4900 species in 938 genera of 40 tribes, is the largest subfamily within Pentatomidae (Eduardo and David 2018). Pentatominae species are phytophagous by sucking the sap from the stems, leaves or fruit. To date, phylogenetic analyses within Pentatominae are still limited and mainly employed morphological data (Filipe et al. 2017). Here we sequenced and annotated the complete mitochondrial genome (mitogenome) of *P. viridissima,* which will be helpful to better understand the diversity and phylogeny of Pentatominae.

Adult specimens of *P. viridissima* were collected from Yuzhong County (104°34′20″E, 36°26′30″N), Gansu Province, China, in July 2018. Samples (voucher number: YZ-7) have been deposited in the State Key Laboratory of Grassland Agro-Ecosystems, College of Pastoral Agricultural Science and Technology, Lanzhou University, Lanzhou, China. The total genomic DNA was extracted from a single specimen using a DNeasy Tissue Kit (Qiagen, German). The *P. viridissima* mitogenome was amplified by using a set of universal and specific primer pairs, and sequenced in both directions.

The complete mitogenome of *P. viridissima* was a circular molecule of 15,118 bp long (GenBank accession number: NC_050166), comprising of 13 protein-coding genes (PCGs), 22 transfer RNA genes (tRNAs), 2 ribosomal RNA unit genes (*rrnL* and *rrnS*) and a large non-coding region (putative control region). The order and orientation of the mitochondrial genes was identical to the inferred ancestral arrangement of insects (Boore 1999). Gene overlaps were found at five gene junctions and involved a total of 26 bp, ranging in size from 1 to 8 bp. The longest overlap (8 bp) existed between *trnW* and *trnC*. A total of 127 bp intergenic spacers were present in sixteen positions, ranging in size from 1 to 27 bp. The longest intergenic spacers (27 bp) existed between *trnS2* and *nad1*.

The nucleotide composition of the *P. viridissima* mitogenome was significantly biased toward A and T, with an A + T content of 76.0% (A = 42.3%, C = 13.5%, G = 10.5%, T = 33.7%). This mitogenome presented a positive AT-skew (0.11) and a negative GC-skew (-0.13) on the J-strand. The *rrnL* was 1,287 bp long with an A + T content of 78.6%, and the *rrnS* was 803 bp with an A + T content of 77.3%, as found in most insect mitogenomes. Among the 13 PCGs, the lowest A + T content was 69.5% in *cox1*, while the highest was 82.7% in *nad6*. Ten PCGs started with a typical ATN codon: two (*cox2*, *nad6*) with ATA, three (*nad2*, *nad5* and *nad4L*) with ATT, four (*atp6*, *cox3*, *nad4* and *cob*) with ATG, one (*nad3*) with ATC. The other two PCGs started with TTG (*atp8*, *nad1*) and the remaining one started with AAC (*cox1*). Nine PCGs terminated with TAA or TAG, whereas four PCGs terminated with an incomplete stop codon T. All of the 22 tRNAs, ranging from 63 bp (*trnS1*) to 75 bp (*trnK*), had a typical cloverleaf structure, except for *trnS1* which lacked the dihydrouridine arm.

Phylogenetic analysis was performed with the concatenated nucleotide sequences of 13 PCGs and 2 ribosomal RNA genes (*rrnS* and *rrnL*) from 17 Pentatominae species and *Cazira horvathi* (Asopinae, outgroup). We conducted Bayesian inference using MrBayes 3.2.6 (Ronquist and Huelsenbeck 2003) on the CIPRES Science Gateway 3.3 (Miller et al. 2010). The Bayesian phylogenetic tree supported that *P. viridissima* clustered with *Nezara viridula* from the same tribe Nezarini, and recovered a phylogeny of Pentatominae: (Menidini + (Strachiini + (Pentatomini + ((Cappaeini + Halyini) + (Eysarcorini + (Nezarini + Carpocori)))))) (Figure 1).

**Figure 1.**
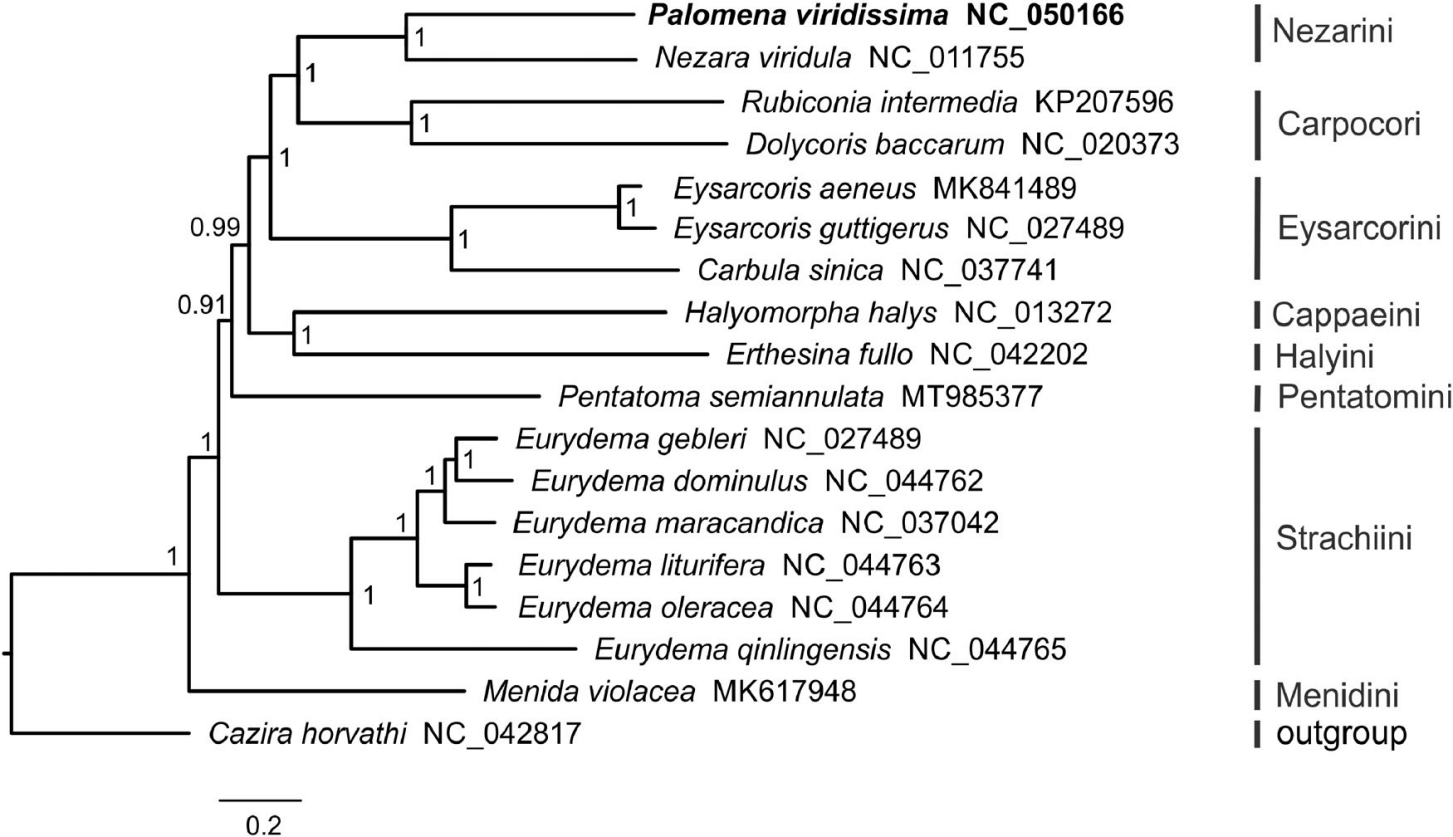
Bayesian phylogenetic tree inferred from the concatenated nucleotide sequences of 13 mitochondrial protein-coding genes and 2 ribosomal RNA genes (*rrnS* and *rrnL*) of 17 Pentatominae. Numbers at the nodes are posterior probabilities.

## Data Availability

The genome sequence data that support the findings of this study are openly available in GenBank of NCBI at (https://www.ncbi.nlm.nih.gov/) under the accession no NC_050166.
